# Early Detection of College Students' Psychological Problems Based on Decision Tree Model

**DOI:** 10.3389/fpsyg.2022.946998

**Published:** 2022-08-10

**Authors:** Yunpeng Huang, Shaoan Li, Bo Lin, Shuai Ma, Jian Guo, Chunli Wang

**Affiliations:** ^1^School of Creative Design, Guilin Institute of Information Technology, Guilin, China; ^2^School of Information Engineering, Guilin Institute of Information Technology, Guilin, China; ^3^School of Tourism Data, Guilin Tourism University, Guilin, China

**Keywords:** ID3 algorithm, Python, SPSS, decision tree model, Kendall correlation coefficient, psychological problems

## Abstract

The paper starts with the research on the early discovery of college students' psychological problems. Besides, it analyzes the data of the general survey of college students' mental health in a certain university, the existing data of students with psychological problems, and the questionnaire data of students' basic information in school. By comprehensively using the decision tree model and Kendall correlation analysis and other methods, using Python and SPSS software to preprocess the data and realize the model, it can obtain a psychological problem prediction model based on the objective behavior data of college students. The model is actually analyzed, and it gets good results.

## Introduction

At present, people's lives are objectively confronted with the contradiction between the growing demand for a better life and the unbalanced and insufficient development. Psychological problems of varying degrees often occur due to pressure from various sources. Through the research on people's mental health status by Chinese scholars in the past 20 to 30 years, and the first blue book on mental health in China, “Report on the Development of Chinese National Mental Health (2017~2018) shows that the psychological problems of different groups in China are increasing day by day.” The report said that 11% had poor mental health and had mild to moderate psychological problems; 2–3% had poor mental health and may have moderate to severe psychological problems. For the college students in our country, this group has just entered adulthood and is about to enter the society. They often face pressure and confusion in terms of study, employment, love, family and other aspects. There is a high chance of lack of self-healing ability in times of stress. In recent years, vicious incidents caused by the mental health problems of college students have emerged one after another. The embarrassing extreme behavior of these college students is a devastating disaster for themselves and their families. It not only affects the normal teaching management of the school and the reputation of teaching and educating people, but also affects the surrounding Classmates, teachers, and friends have serious psychological burdens, and they will spend a lot of time, energy, and personal wealth in the school to deal with crisis emergencies, and may become one of the sources of social disharmony and instability.

Therefore, in the management of college students, it is necessary to pay attention to the construction and improvement of the early warning mechanism for high-risk groups of psychological problems for college students. It actively does a good job in the prevention and intervention of high-risk groups of psychological problems. It is of great significance to enhance the physical and mental health of college students and promote the construction of a harmonious society (Pang, 2016).

The United States is the first country to pay attention to the psychological problems of college students and establish a psychological counseling center. In the 1870s, the University of Pennsylvania established the first psychological counseling clinic (Fayyad et al., 1996), which realized the combination of education and psychology, and enabled college students to pass the knowledge of psychology. If they come to receive mental health education, psychologists and students will become good teachers and friends. Psychologists discover the psychological problems of students in the process of communicating with students, so as to carry out psychological intervention. For the first time, it classifies different ages, genders and races to carry out targeted psychological. The research established a psychological book corner and a psychological counseling station for classmates, which greatly reduced accidents caused by psychological problems (Zhao, 2020).

Compared with developed countries, domestic college students' psychological research is relatively late. At the beginning of the 20th century, my country's colleges and universities achieved certain results in the early warning and intervention of college students' psychological crisis (Cheng, 2018). Guo Lan and Gong Yu established a psychological intervention system in “Essential Crisis Prevention and Crisis Beyond Crisis—Construction and Operation of College Students' Psychological Crisis Early Warning System” (Guo and Gong, 2008). Gao Lifang's “On the Construction of the Management Mechanism of College Students' Psychological Crisis Early Warning” (Gao, 2014) proposed the construction of a psychological intervention system at the home and school levels. Liu Jing's “Psychological Crisis Intervention of College Students and College Students' Psychological Archives” (Liu, 2015) greatly improved the real-time nature of psychological crisis intervention by constructing personal psychological archives.

At present, major colleges and universities basically have their own college students' mental health assessment system. After the college students' mental health assessment work is carried out every year, a large amount of data will be generated. Precise and effective psychological counseling and intervention is very likely to save a young life. However, due to the obvious discrepancy between the predicted results obtained by the general survey questionnaire on college students' mental health and the actual situation, the paper redesigned the questionnaire, and selected the decision tree classification algorithm in data mining (Sun, 2020) according to the actual situation of the university. The students' basic information questionnaire data in colleges and universities were analyzed and a mental health early warning model was obtained. According to the combination of ID3 algorithm and decision tree model, early detection and effective intervention of psychological problems of college students can be achieved to prevent the occurrence of tragedies caused by psychological problems of college students (Zhang et al., 2008, 2011).

## Data Selection and Pre-Processing

### Research Ideas

Firstly, it cleaned the missing and wrong data in the questionnaire data set. Then the attributes of the data set are assigned. Finally, the data is screened twice. Firstly, time removes the attributes that are weakly related to this research; Secondly, according to the Kendall correlation analysis and the significant attributes of this study were incorporated into the decision tree model (Wu et al., 2011).

### Analysis of the General Survey Data of College Students' Mental Health

The school has been using the general psychological health survey questionnaire for college students for analysis. According to the results of the data, students have psychological problems to talk to, so as to find out students with psychological problems and intervene. In the questionnaire, there are reasons such as avoidance, self-protection, perfunctory answering, and subjective judgment bias, resulting in a low degree of overlap between students who actually show psychological problems and those selected by the questionnaire, so it is necessary to redesign a set of questionnaires. The questions of the questionnaire are mostly direct questions about the emotional state of the respondents. In order to avoid inaccurate and ambiguous answers due to problems such as strong subjectivity and poor quantification, the types of questions involved in the new questionnaire should focus on students' objective information, behavioral performance and quantifiable principles, and the prediction model established based on reliable data research is imminent to warn college students' psychological problems (Wen et al., 2021).

### Research Objects

The data set of this research is collected from all students of the 2018–2021 design school of a university in Guilin. The data were obtained through psychological questionnaires organized by counselors in class meetings (Zhou and Guo, 2013; Xin et al., 2019). The specific information is as follows.

**Figure 1:**
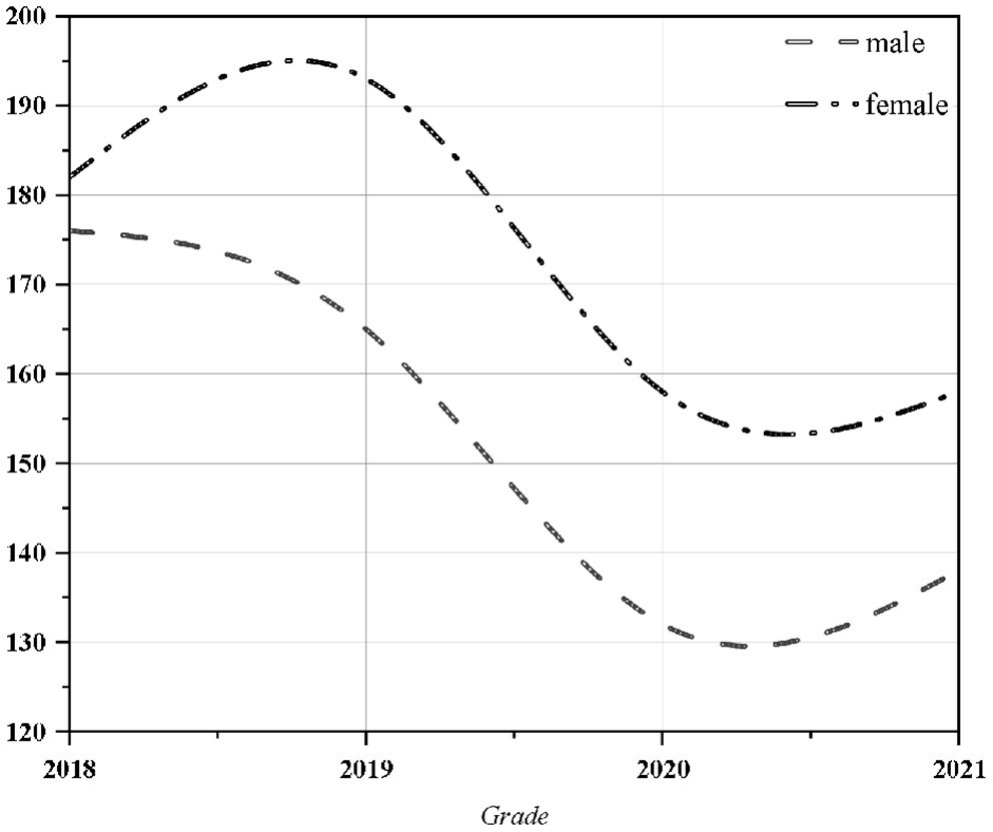
School of design student questionnaire information.

Statistics of the four grades of the School of Design were collected. As can be seen from the Figure 1, a total of 1,302 valid questionnaires were received, including 611 questionnaires for boys and 691 questionnaires for girls more balanced.

### Data Collection

After the data collection is completed, in order to facilitate the research, we have made custom assignments to the 30 variables in the following table (Yu and Wang, 2019), as shown in Table 1.

**Table 1:** Questionnaire attribute assignment table.

	**Variable**	**Assignment**
1	Gender	Female = 0, male = 1
2	Year	Freshman = 1, Sophomore = 2 Junior = 3, Senior = 4
3	Is it a student cadre?	Yes = 0, No = 1
4	Have you ever received an award during college?	Yes = 0, No = 1
5	No punishment during college	No = 0, Yes = 1
6	Your academic performance is	good = 0, average = 1, poor = 2
7	What do you think your personality traits tend to be?	More extroverted = 0, more introverted = 1
8	What is the average number of requests for leave during the semester in college?	Less than 3 times = 0, more than 3 times = 1
9	What are the types of leave?	More personal leave = 0, more sick leave = 1
10	Family composition	Single parent = 0, double parent = 1, orphan = 2, divorced parent = 3
11	Is it an only child?	Yes = 0, No = 1
12	How is the family economy	generally = 0, rich = 1, poor = 2
13	What is the relationship between family members?	Generally = 0, disharmony = 1, harmony = 2
14	The degree of self-perceived learning pressure during university is relatively	small = 0, generally = 1, and relatively high = 2
15	Has there been or is a major family emergency	No = 0, Yes = 1
16	Whether there is domestic violence among family members	Never = 0, Ever = 1, Always =2
17	Are you physically disabled?	No = 0, Yes = 1
18	Do you have any medical history?	No = 0, Yes = 1
19	No history of taking medication since	No = 0, Ever = 1, Always = 2
20	Have you ever experienced school bullying?	No = 0, ever = 1, always = 2
21	Has there been any conflict between teachers and students?	No = 0, once = 1, always = 2
22	Average absenteeism during the semester in college	No = 0, Occasionally = 1, Frequent = 2
23	Have you ever taken a leave of absence from school?	No = 0, Yes = 1
24	Average participation in various group activities during the semester in college	More than 3 times = 0, <3 times = 1, never participating = 2
25	Self-employment pressure	generally = 0, less = 1, greater = 2
26	Personal hygiene status	Don't care = 0, generally = 1, very important = 2
27	Contradictions in the dormitory	Good=0, Fair=1, Poor=2
28	Self-eating rules	Good = 0, Average = 1, Poor = 2
29	Are you keen on computer games?	Dislike = 0, generally = 1, like = 2
30	Self-sleep quality	Good = 0, average = 1, poor = 2
	Psychological situation	Yang 1 if there is a psychological problem, and Yin 0 if there is no psychological problem

### Data Selection

In the questionnaires filled in, there are many attributes that are weakly related to this research. According to basic common sense and searching for relevant information, it deletes irrelevant factors such as class, student number, name, ethnicity, etc. Then it confirms the remaining attributes and Del (Kendall) correlation analysis (Zhang et al., 2000).

Using statistical analysis software *spss*, it selects Kendall correlation analysis, and gets the results as shown in Table 2.

**Table 2:** Kendall analysis results.

**Number**	**Influencing variable**	**Correlation coefficient**	***P*-value**
1	Academic performance	0.073	0.008
2	Number of leave requests	0.085	0.002
3	Is there a medical history	0.095	0.001
4	Whether there is a history of taking medication	0.151	0.000
5	Have you ever experienced school bullying?	0.070	0.012
6	Whether there is a leave of absence experience	0.065	0.019
7	Own diet	0.090	0.001
8	Own sleep quality	0.094	0.000

According to the results of Kendall's correlation analysis (Zhang et al., 2000; Li et al., 2008), “Academic Performance,” “Number of Leave Requests,” “whether there is a Medical History,” “whether there is a history of taking drugs,” “whether you have experienced School Bullying,” “whether you have The *p*-values of “Experience of School Leave,” “Self-eating Pattern,” and “Self-sleep Quality” were all <0.01 (Xin et al., 2012), which was why they were included in the decision tree model. According to my country's 2020 Blue Book on Mental Health, “Report on the Development of Chinese National Mental Health (2019–2020),” nearly 20% of college students have different degrees of psychological problems. For this reason, we assign scores to the questionnaire. After sorting, in addition to students with actual psychological problems as positive samples, the top 20% of the data samples are taken as positive cases to construct the model (Wang et al., 2000; Xin et al., 2012).

**Figure 2:**
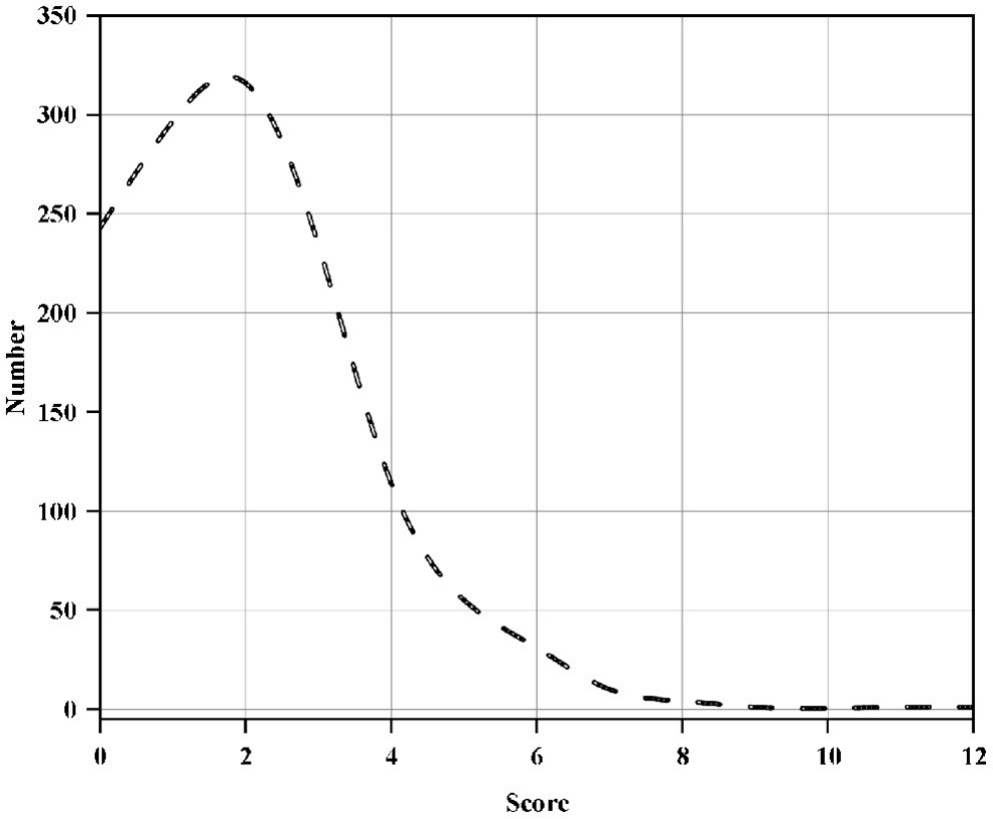
Student psychological score pie chart.

The psychological scores of the students are shown in the Figure 2.

As can be seen from the above Figure 2, the highest score of students' psychology is 12 points, and the lowest score is 0 points. It is reasonable to take 4 points as the sample boundary. Therefore, students with actual psychological problems are excluded as positive samples, and students with psychological scores >4 points are set. It sets as a positive case, and construct a decision tree model.

## III. Decision Tree - Early Warning Model of College Students' Psychological Crisis

### Research Ideas

**Figure 3:**
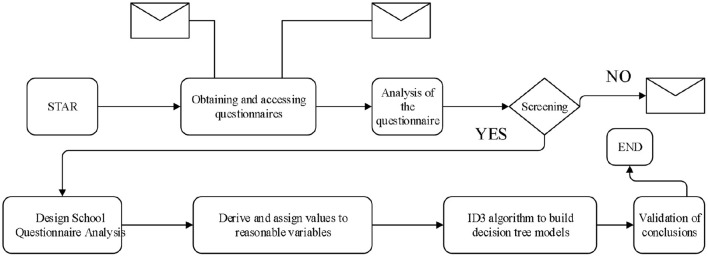
Research idea flow chart.

Firstly, through designing the questionnaire (Zhang, 2012) of the department, it uses the ID3 algorithm, the decision tree model is implemented to obtain the psychological problem data. Then the conclusion is verified according to the questionnaires of the students of the information department of the school. The specific implementation process is shown in Figure 3.

### Related Concepts

Decision tree is a basic classification and regression method (Zhang, 2012). We mainly use decision trees for classification. The decision tree model has a tree structure, and in classification problems (Ma, 2019), it represents the process of classifying instances based on features. When learning, use the training data to build a decision tree model. When predicting, the new data is classified by using the established decision tree model. The ideas of these decision tree learning mainly include ID3 algorithm (Qu et al., 2003), C4.5 algorithm (Li et al., 2013) and CART algorithm (Chen and Xia, 2011).

The algorithm of decision tree learning is usually a process of recursively selecting the optimal feature and dividing the training data according to the feature, so that each sub-data set has a best classification process. This process corresponds to the division of the feature space and the construction of the decision tree. Firstly, it builds the root node, selects the optimal feature of all training data, and divides the dataset into subsets according to this feature. So that each subset has a best classification under the current conditions; if it has been basically correctly classified, then build a leaf node divide these subsets to the corresponding leaf nodes; if there are still subsets that are not correctly classified, select new optimal features for them and continue to divide; then build corresponding nodes; until all training data subsets are correctly classified or end without the proper feature. Finally, a decision tree can be generated.

### Decision Tree Splitting Based on ID3 Algorithm

ID3, C4.5, and CART algorithms are the most common used algorithms in decision tree algorithms. For this article, we study mental health-related data. Due to the large number of discrete data and the small amount of attribute values, comprehensive consideration, the ID3 algorithm was chosen to classify this study. In the ID3 algorithm, the decision tree is constructed recursively by selecting features corresponding to the information gain criterion on each node. The process starts from the root node, calculates the information gain of all possible features, and selects the feature with the largest information gain as the node feature; then establishes child nodes with different values of this feature, and then uses the recursive method to call the above method to construct a complete the decision tree (Zhang and Ning, 2015).

In constructing a decision tree, it is necessary to determine its split nodes, and the determination of split nodes needs to be divided by certain rules and attributes. In the ID3 algorithm, the attribute we refer to is mainly information gain.

Assuming that *K* is a training sample set, this sample set contains *n* samples of categories, then *n* different classes *C*_*i*_ (*i* = 1, 2, 3…, n) can be defined, and the probability of *C*_*i*_ is represented by *p*_*i*_. The following formula can be derived:


(1) $$\begin{array}{l} Info\left(K\right)=-\overset{n}{\underset{i=0}{\sum }}p_{i}\;\log_{2}\;p_{i} \end{array}$$

*Info*(*K*) is called entropy, it describes the purity of any sample set. Entropy can represent uncertainty. The greater the uncertainty of the variable, the greater the entropy value. The value of entropy ranges from 0 to 1.

Assuming that the *K* sample of is divided according to attribute A, then there *K*_*j*_ is the *j*th subset divided according to attribute A, then the following formula is obtained:


(2) $$\begin{array}{l} Info_{A}\left(K\right)=-\overset{m}{\underset{j=1}{\sum }}\frac{\left|K_{j}\right|}{\left|K\right|}Info_{A}\left(K_{j}\right) \end{array}$$

*Info*_*A*_(*K*) called sample entropy, it expresses the expected information for dividing K based on attribute A.

Through the above two formulas, its information gain can be calculated, and the formula is as follows:


(3) $$\begin{array}{l} Gain\left(K,A\right)=Info\left(K\right)-Info_{A}\left(K\right) \end{array}$$

Gain(K,A) is for the information gain, it also known as the amount of information acquisition (Information Gain). Information gain is the information about the value of the objective function due to the value of a given attribute (Li et al., 2005).

In the process, we select the test attribute with the largest information gain as the root node of the decision tree. Then it generates the first decision tree, and then recursively perform the above process for each leaf node. Finally, it obtains a complete classification decision tree.

### The Process of Building a Decision Tree

To build a psychological crisis prediction model, from the data screened above, there are 8 attributes to be added to the decision tree, namely academic performance, number of leave requests, medical history, medication history, campus bullying, school leave experience, eating patterns, sleep quality, and the value of its properties. The final dataset is used for model training. To keep the results clean and uncluttered, start by naming the eight attributes of its dataset with abbreviations, as show in Table 3.

**Table 3:** Variable symbol description.

	**Property**	**Abbreviation**
1	Academic performance	CJ
2	Number of leave requests	QJ
3	Medical history	BS
4	Medication history	FYS
5	School bullying	BL
6	Leave of absence	XX
7	Diet	YS
8	Sleep quality	SM

In the obtained training set samples *K*, we pass the real results and add the assigned values, a total of 222 are positive, and 1,085 are negative, so the entropy of the training set is calculated as:


(4) $$\begin{array}{c} Info\left(K\right)=-\frac{222}{1307}\log_{2}\frac{222}{1307}-\frac{1085}{1307}\log_{2}\frac{1085}{1307}\\ =0.6573721232057073 \end{array}$$

The sample entropy and information gain corresponding to the attributes of academic performance, number of leave requests, medical history, medication history, school bullying, school leave experience, dietary pattern, and sleep quality are calculated below (Li and Xin, 2008).

In terms of academic performance, there are three attribute values 0, 1, and 2, and the sample data are 1,021, 258, and 28, respectively. There are 123 positives and 898 negatives of psychological crisis with attribute value 0; 83 positives and 175 negatives with attribute value 1; 16 positives and 12 negatives with attribute value 2. It calculates its sample entropy as:


(5) $$\begin{array}{l} Info_{CJ}\left(K\right)=\frac{1021}{1307}\left(-\frac{123}{1021}\log_{2}\frac{123}{1021}-\frac{898}{1021}\log_{2}\frac{898}{1021}\right)\\ \;+\frac{258}{1307}\left(-\frac{83}{258}\log_{2}\frac{83}{258}-\frac{175}{258}\log_{2}\frac{175}{258}\right)\\ \;+\frac{28}{1307}\left(-\frac{16}{28}\log_{2}\frac{16}{28}-\frac{12}{28}\log_{2}\frac{12}{28}\right)\\ \;=0.6145741784450385 \end{array}$$

The information gain of academic performance is as follows:


(6) $$\begin{array}{l} Gain\left(CJ\right)=Info\left(K\right)-Info_{CJ}\left(K\right)=0.04279794476066878 \end{array}$$

Through the same steps above, we can finally calculate the information gain of the eight attributes:


(7) $$\begin{array}{l} Gain\left(CJ\right)=0.04279794476066878 \end{array}$$

(8) $$\begin{array}{l} Gain\left(QJ\right)=0.04692457281442097 \end{array}$$

(9) $$\begin{array}{l} Gain\left(BS\right)=0.10178147259099068 \end{array}$$

(10) $$\begin{array}{l} Gain\left(FYS\right)=0.13075980120050001 \end{array}$$

(11) $$\begin{array}{l} Gain\left(BL\right)=0.07277279223941935 \end{array}$$

(12) $$\begin{array}{l} Gain\left(XX\right)=0.01172718186654953 \end{array}$$

(13) $$\begin{array}{l} Gain\left(YS\right)=0.1192610978220714 \end{array}$$

(14) $$\begin{array}{l} Gain\left(SM\right)=0.12125218656041414 \end{array}$$

**Figure 4:**
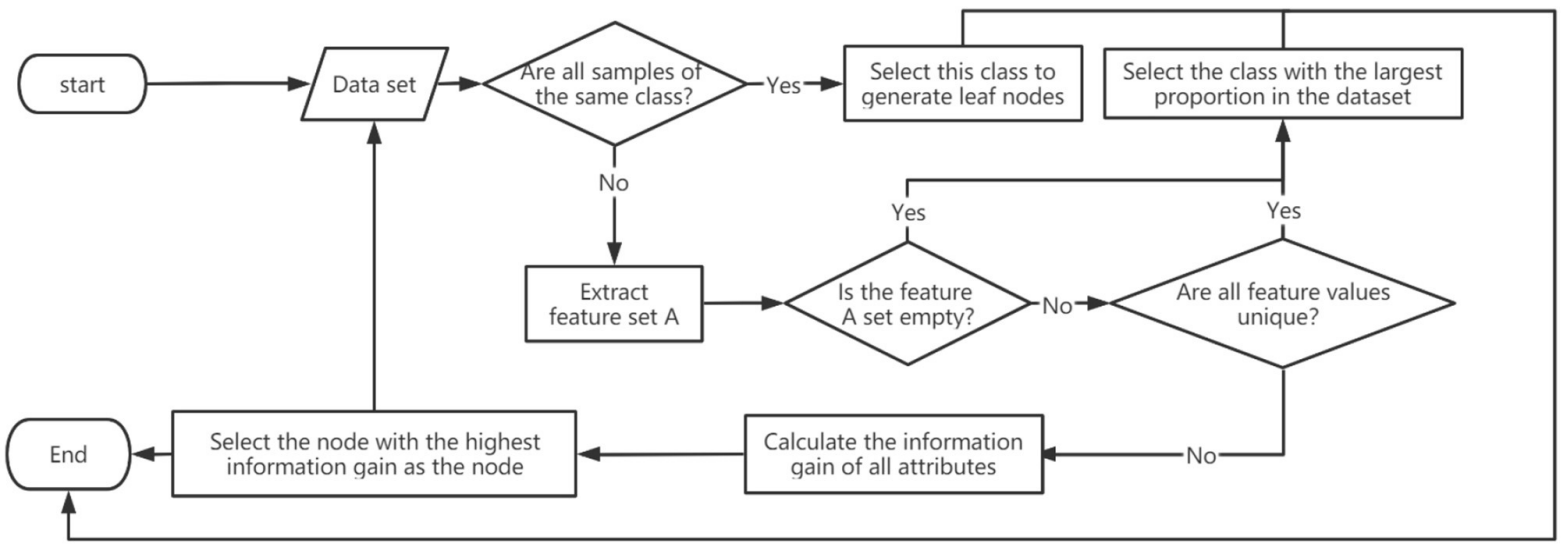
ID3 algorithm flowchart.

From the above calculation results, it can be seen that the information gain of whether there is a medication history is the largest among all attributes, so the presence or absence of medication history is selected as the test attribute of the root node, and then the above process is recursively used for each node to generate the final decision tree (Gao et al., 2008) in the following Figure 4.

**Figure 5:**
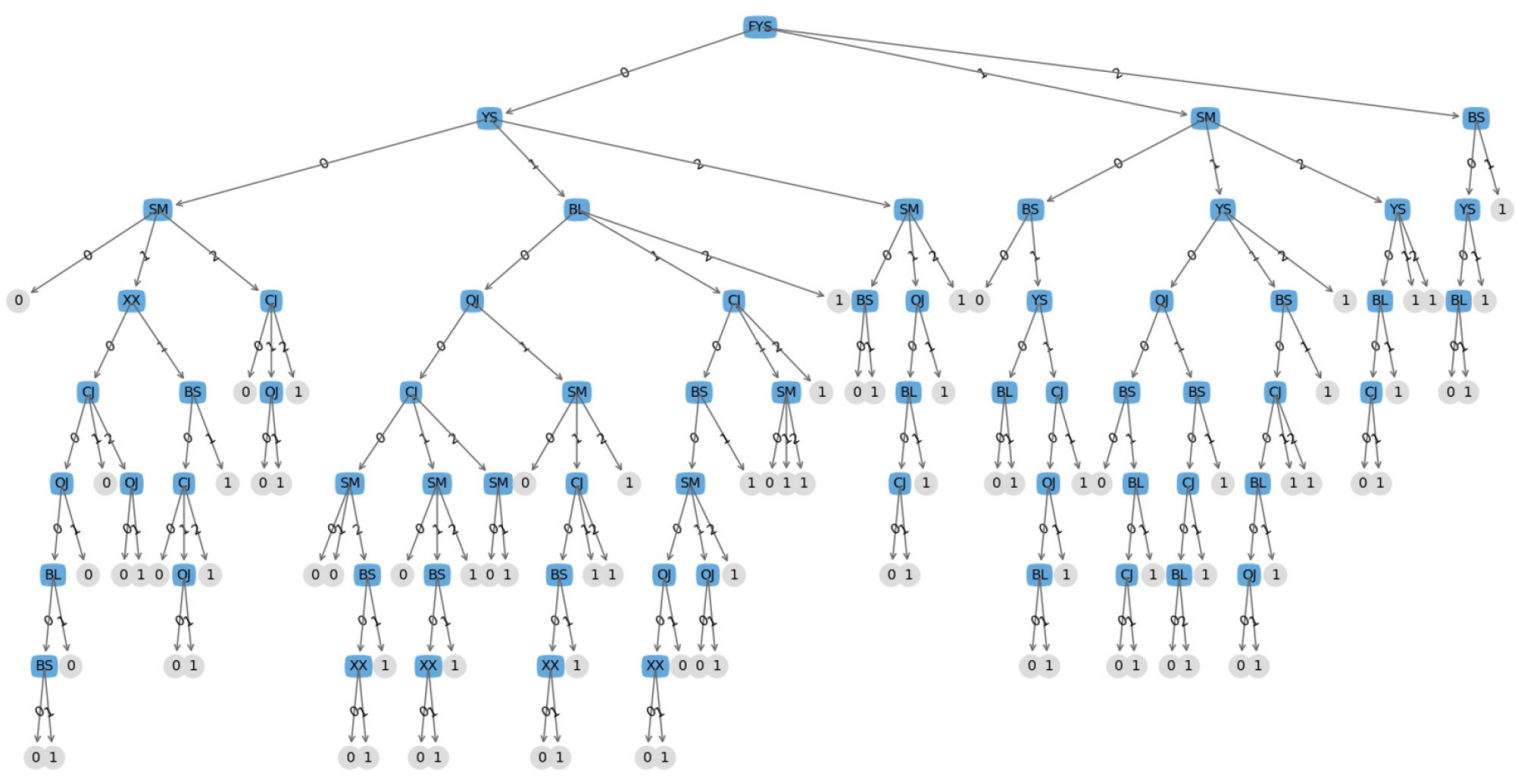
Decision tree model diagram.

We use the ID3 algorithm to split the decision tree. Firstly, we filter the input data set, calculate the information gain of all attributes. Finally, we select the node with the highest information gain as the node, and continue the process to generate a decision tree. After processing the data and constructing a decision tree (Fan, 2004), and visualizing the obtained decision tree through machine learning actual combat codes are as show in Figure 5.

### Result Acquisition

According to the above process and the classification decision tree model, we extract the data with positive results. That is, the data that is finally judged to have a psychological crisis as show in Tables 4–7.

**Table 4:** Psychological crisis judgment Table 1.

**Number**	**Rule**
1	Medication history: none, dietary pattern: good, sleep quality: average, school leave experience: no, academic performance: good, number of leave requests: <3 times, school bullying: no, medical history: yes
2	Medication history: none, dietary pattern: good, sleep quality: average, school leave experience: no, academic performance: poor, number of leave requests: more than 3 times
3	Medication history: none, eating pattern: good, sleep quality: average, school leave experience: yes, medical history: no, academic performance: average, number of leave requests: more than 3 times
4	Medication history: none, eating pattern: good, sleep quality: average, school leave experience: yes, medical history: no, academic performance: poor
5	Medication history: none, dietary pattern: good, sleep quality: average, school leave experience: yes, medical history: yes
6	6 Medication history: none, dietary pattern: good, sleep quality: poor, academic performance: average, number of leave requests: more than 3 times
7	Medication history: none, eating pattern: good, sleep quality: poor, academic performance: poor
8	Medication history: none, dietary pattern: general, school bullying: none, number of leave requests: <3 times, academic performance: good, sleep quality: poor, medical history: no, school leave experience: yes
9	9 Medication history: none, dietary pattern: general, school bullying: none, number of leave requests: <3 times, academic performance: good, sleep quality: poor, medical history: yes
10	Medication history: no, dietary pattern: general, school bullying: no, number of leave requests: <3 times, academic performance: general, sleep quality: general, medical history: no, school leave experience: yes

**Table 5:** Psychological crisis judgment Table 2.

**Number**	**Rule**
11	Medication history: none, dietary pattern: normal, school bullying: none, number of leave requests: <3 times, academic performance: normal, sleep quality: normal, medical history: yes
12	Medication history: none, dietary pattern: general, school bullying: none, number of leave requests: <3 times, academic performance: average, sleep quality: poor
13	Medication history: none, dietary pattern: general, school bullying: none, number of leave requests: <3 times, academic performance: poor, sleep quality: average
14	Medication history: none, dietary pattern: general, school bullying: no, number of leave requests: more than 3 times, sleep quality: general, academic performance: good, medical history: no, school leave experience: yes
15	Medication history: none, dietary pattern: normal, school bullying: none, number of leave requests: more than 3 times, sleep quality: normal, academic performance: good, medical history: yes
16	Medication history: none, dietary pattern: average, school bullying: none, number of leave requests: more than 3 times, sleep quality: average, academic performance: average or poor
17	Medication history: none, dietary pattern: general, school bullying: ever, academic performance: good, medical history: no, sleep quality: good, number of leave requests: <3 times, school leave experience: yes
18	Medication history: none, dietary pattern: general, school bullying: ever, academic performance: good, medical history: no, sleep quality: average, number of leave requests: more than 3 times
19	Medication history: none, dietary pattern: general, school bullying: ever, academic performance: good, medical history: no, sleep quality: poor
20	Medication history: none, dietary pattern: general, school bullying: yes, academic performance: good, medical history: yes

**Table 6:** Psychological crisis judgment Table 3.

**Number**	**Rule**
21	Medication history: none, dietary pattern: average, school bullying: ever, academic performance: average, sleep quality: average or poor
22	Medication history: none, dietary pattern: general, school bullying: ever, academic performance: poor
23	Medication history: none, dietary pattern: general, school bullying: always
24	Medication history: none, dietary pattern: poor, sleep quality: good, medical history: yes
25	Medication history: none, dietary pattern: poor, sleep quality: average, number of leave requests: <3 times, school bullying: none, academic performance: average
26	Medication history: none, dietary pattern: poor, sleep quality: average, number of leave requests: <3 times, school bullying: ever
27	Medication history: none, dietary pattern: poor, sleep quality: average, number of leave requests: more than 3 times
28	Medication history: none, dietary pattern: poor, sleep quality: poor
29	Medication history: yes, sleep quality: good, medical history: yes, dietary pattern: good, school bullying: yes
30	Medication history: ever, sleep quality: good, medical history: yes, dietary pattern: average, academic performance: good, number of leave requests: <3 times, school bullying: ever

**Table 7:** Psychological crisis judgment Table 4.

**Number**	**Rule**
31	Medication history: ever, sleep quality: good, medical history: yes, dietary pattern: average, academic performance: good, number of leave requests: more than 3 times
32	Medication history: ever, sleep quality: good, medical history: yes, dietary pattern: average, academic performance: average
33	Medication history: ever, sleep quality: average, dietary pattern: good, number of leave requests: <3 times, medical history: yes, school bullying: no, academic performance: good
34	Medication history: yes, sleep quality: average, dietary pattern: good, number of leave requests: <3 times, medical history: yes, school bullying: yes
35	Medication history: ever, sleep quality: average, dietary pattern: good, number of leave requests: more than 3 times, medical history: no, academic performance: good, school bullying: ever
36	Medication history: ever, sleep quality: average, dietary pattern: good, number of leave requests: more than 3 times, medical history: no, academic performance: average
37	Medication history: ever, sleep quality: average, dietary pattern: good, number of leave requests: more than 3 times, medical history: yes
38	Medication history: ever, sleep quality: average, dietary pattern: average, medical history: no, academic performance: good, school bullying: no, number of leave requests: more than 3 times
39	Medication history: ever, sleep quality: average, dietary pattern: average, medical history: no, academic performance: good, school bullying: ever
40	Medication history: ever, sleep quality: average, dietary pattern: average, medical history: no, academic performance: average or poor

## Model Validation

**Figure 6:**
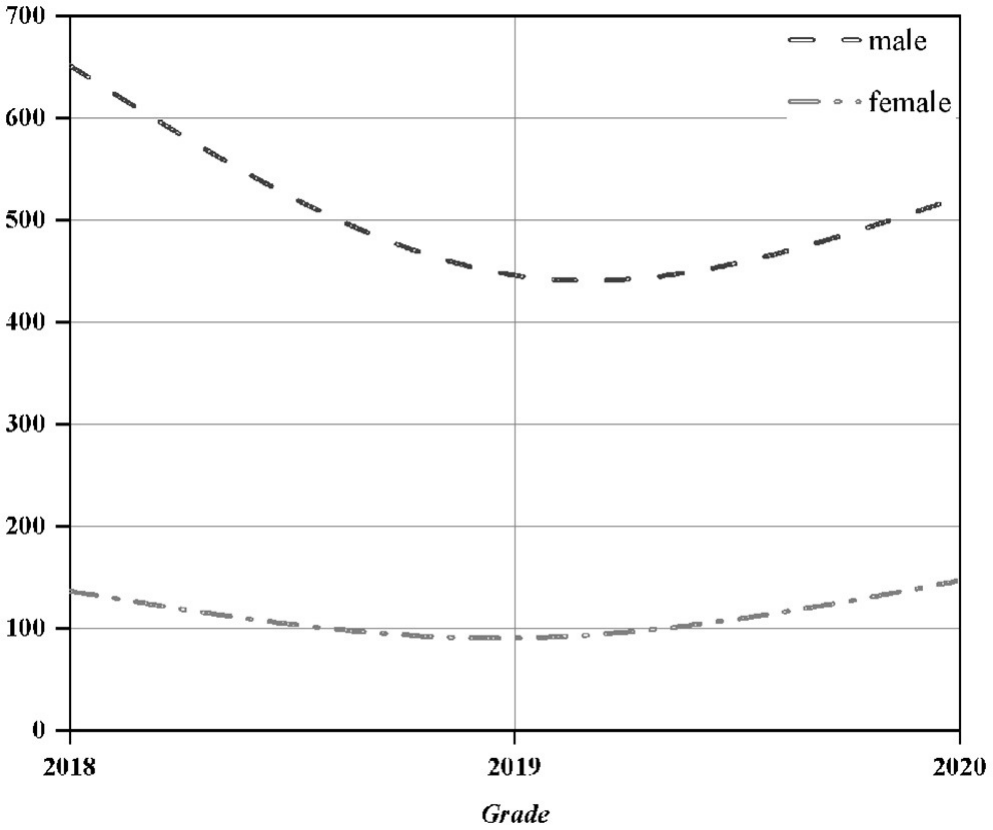
Information on the student questionnaire of the school of information.

Through the construction of the above decision tree, a prediction model about the psychological problems of college students is obtained. The main conclusion is 48 pieces of psychological crisis data. In order to verify the practicability and accuracy of the conclusions, the information of all students in the School of Information Engineering of the school was collected to verify the model. The specific data are shown in Figure 6.

The School of Information has collected statistics on the questionnaires of students in three grades. It received a total of 1,950 valid questionnaires, including 1,581 for boys and 369 for girls. The ratio of male to female is in line with the normal ratio of male to female in science and engineering. Among them, psychological events or psychological problems have actually occurred. There are 19 students, and the proportion of males and females in the three grades is similar, which is more suitable as a verification sample.

Through data screening, all the data items in the questionnaire for the model were screened out, including options for eight groups of questions: academic performance, number of leave requests, medical history, medication history, school bullying, school leave experience, eating patterns, and sleep quality. By comparing the 48 psychological crisis data options obtained by the decision tree with the questionnaire data options of these 1,981 students, we finally came to the following conclusions.

The total number of students participating in the survey was 1,981, and 261 were found to need psychological warning through comparison. Among these 261 people, 10 people have actually experienced psychological problems or psychological events, accounting for about 53% of the actual psychological problems or psychological events. Therefore, the model constructed by the decision tree has good accuracy and generality (Yao and Liu, 2008).

## Conclusion

In response to the early discovery of college students' psychological problems, we conducted correlation analysis on the results of the questionnaire, and screened out the attributes that have greater correlation with psychological crisis. Then we used the decision tree-based analysis of the results of the questionnaire to find out the results of academic performance, asking for leave the general relationship between the number of times, medical history, medication history, school bullying, school leave experience, dietary patterns, sleep quality and psychological crisis problems, we can use this questionnaire to understand the students, through the classification decision tree obtained 48 items the psychological crisis conclusion was used to screen out the students who need to focus on and understand. We obtained a psychological problem prediction model based on the objective behavior data of college students. The model was actually verified and analyzed, and the obtained 48 students were classified as possible psychological problems. Or students who may have a crisis event, and establish a file of students with psychological problems, improve the accuracy and effectiveness of psychological early warning, and prevent college students from tragedies due to lack of timely intervention for mental health problems.

## Data Availability Statement

The datasets presented in this article are not readily available because It's confidential. Requests to access the datasets should be directed to yunpenghuang3207@sina.com.

## Author Contributions

YH: conceptualization, methodology, software, investigation, formal analysis, and writing—original draft. SL: data curation and writing—original draft. BL: visualization, investigation, and software. SM: resources, supervision, and validation. JG: visualization and writing—review and editing. CW: conceptualization, funding acquisition, resources, supervision, and writing—review and editing. All authors contributed to the article and approved the submitted version.

## Funding

The work was supported by 2021 Ministry of Education Humanities and Social Sciences Research Special Task Project (21JDSZ3050), Nature and Science Foundation of Anhui (2008085QA08), and 2019 Project of Foundational Research ability enhancement for Young and Middle-aged University Faculties of Guangxi (2019KY1046).

## Conflict of Interest

The authors declare that the research was conducted in the absence of any commercial or financial relationships that could be construed as a potential conflict of interest.

## Publisher's Note

All claims expressed in this article are solely those of the authors and do not necessarily represent those of their affiliated organizations, or those of the publisher, the editors and the reviewers. Any product that may be evaluated in this article, or claim that may be made by its manufacturer, is not guaranteed or endorsed by the publisher.

## References

[B1] Chen H. L. Xia D. X. (2011). Application research of data mining algorithm based on CART decision tree. Coal Technol. 30, 164–166.

[B2] Cheng F. Y. Li J. L. Yu W. Y. Zhang M. Q. (2018). Application and prospect of data mining technology in the field of psychology, in Chinese Psychological Association Abstracts of the 21st national psychological academic conference (Beijing: Chinese Psychological Society), 1178–1179.

[B3] Fan B. (2004). The application of E-R model in the analysis and design of accounting information system. China Account. Computeriz. 2004, 16–18. 10.3969/j.issn.1673-0194.2004.04.008

[B4] Fayyad U. Piatetsky-Shapiro G. Smyth P. (1996). The kdd process for extracting useful knowledge from volumes of data. Commun. ACM 39, 27–34. 10.1145/240455.240464

[B5] Gao L. F. (2014). On the construction of the management mechanism of college students' psychological crisis early warning. Educ. Res. 2, 84–86.

[B6] Gao Y. Liu D. Y. Qi H. Liu H. (2008). A semi-supervised K-means multi-relational data clustering algorithm. J. Softw. 2008, 2814–2821. 10.3724/SP.J.1001.2008.02814

[B7] Guo L. Gong Y. (2008). Emphasizing crisis, preventing crisis and going beyond crisis: construction and operation of college students' psychological crisis early warning system. Hubei Soc. Sci. 2008, 175–177.

[B8] Li C. Xin L. (2008). Research on the evaluation method of the reliability and validity of the questionnaire. China Health Stat. 2008, 541–544.

[B9] Li D. G. Miao D. Q. Yu B. (2005). Research and improvement of decision tree pruning algorithm. Comput. Eng. 2005, 19–21.

[B10] Li J. S. Li J. H. Liu X. N. Shen X. P. Mi Y. J. (2008). The application of Kendall's W analysis method in medical data processing and the realization method in SPSS. Modern Prev. Med. 2008, 33–42.

[B11] Li X. W. Chen F. C. Li S. M. (2013). Improved C4.5 decision tree algorithm based on classification rules. Comput. Eng. Design 34, 4321–4325+4330. 10.16208/j.issn1000-7024.2013.12.06020980134

[B12] Liu J. (2015). College students' psychological crisis intervention and college student' psychological archives. Res. Explor. 6, 26–28.

[B13] Ma C. (2019). An empirical study on the mental health level of“Post-00” college students-based on the data analysis of nearly 20, 000 2018 Freshmen. Ideol. Theor. Educ. 2019, 95–99. 10.16075/j.cnki.cn31-1220/g4.2019.03.017

[B14] Pang W. (2016). Comparative study on mental health education of Chinese and American college students (Master Thesis). Jilin: Jilin University.

[B15] Qu K. S. Cheng W. L. Wang J. H. (2003). An improved algorithm of ID3 algorithm. Comput. Eng. Applic. 2003, 104–107.

[B16] Sun W. (2020). Research on the Application of Decision Tree Technology in Mental Health Assessment of College Students. Zhengzhou: Zhengzhou University. 10.27466/d.cnki.gzzdu.2020.000560

[B17] Wang L. Zheng X. Su Y. L. (2000). Experimental research on mental health education of teachers and students in higher education. Psychol. Sci. 2000, 297–300+381–382. 10.16719/j.cnki.1671-6981.2000.03.010

[B18] Wen Z. L. Fang J. Shen J. Q. Tan Y. T. Li D. X. Ma Y. M. (2021). Review of domestic psychological statistical methods in 20 years in the new century. Adv. Psychol. Sci. 29, 1331–1344. 10.3724/SP.J.1042.2021.01331

[B19] Wu X. G. Zhou P. Peng W. H. (2011). Application of decision tree algorithm in mental health evaluation of college students. Comput. Applic. Softw. 28, 240–244.

[B20] Xin S. F. Shi M. Zhang F. W. (2019). A cross-sectional historical study on the change of suicidal attitude of Chinese College Students. Chin. J. Clin. Psychol. 27, 401–405. 10.16128/J.CNKI.1005-3611.2019.02.039

[B21] Xin Z. Q. Zhang M. He L. (2012). A cross-sectional historical study on the change of mental health of college students. Acta Psychol. Sin. 44, 664–679. 10.3724/SP.J.1041.2012.00664

[B22] Yao B. Liu R. (2008). Problems and countermeasures in the practice of peer psychological counseling in colleges and universities. Educ. Explor. 2008, 126–127.

[B23] Yu X. Wang W. N. (2019). The application of four-level psychological network in psychological crisis intervention in colleges and universities. J. Shaanxi Radio Telev. Univ. 21, 27–29.

[B24] Zhang D. J. (2012). An integrated study of adolescent mental health and mental quality training. Psychol. Sci. 35, 530–536. 10.16719/j.cnki.1671-6981.2012.03.001

[B25] Zhang D. J. Feng Z. Z. Guo C. Chen X. (2000). Several issues on the study of students' psychological quality. J. Southwest Normal Univ. 2000, 56–62. 10.13718/j.cnki.xdsk.2000.03.012

[B26] Zhang F. H. Fang L. T. Gao P. (2008). Research on psychological crisis and its intervention. World Sci. Technol. Res. Dev. 30, 504–508. 10.16507/j.issn.1006-6055.2008.04.026

[B27] Zhang J. Jing J. Wu X. Y. (2011). A sociological analysis of the declining trend of suicide rate in China. Chin. Soc. Sci. 5, 97–113.

[B28] Zhang L. Ning Q. (2015). Two improvements and applications of CART decision tree. Comput. Eng. Design 36, 1209–1213. 10.16208/j.issn1000-7024.2015.05.018

[B29] Zhao D. (2020). Research and Application of Psychological Crisis Early Warning Model for College Students Based on Decision Tree. Beijing: Beijing Forestry University. 10.26949/d.cnki.gblyu.2020.000184

[B30] Zhou C. Y. Guo Y. Y. (2013). The influence of family social stratum on the mental health of College Students: the mediating effect of the belief of just world. Chin. J. Clin. Psychol. 21, 636–640. 10.16128/J.CNKI.1005-3611.2013.04.017

